# Risk factors and early onset predictors of contralateral hip fractures following initial treatment of the other hip fracture

**DOI:** 10.1186/s12891-026-10160-0

**Published:** 2026-07-02

**Authors:** Yuichiro Ukon, Hirokazu Mae, Akitomo Inoue, Yuya Kanie, Tetsuro Tani, Kazuma Takashima, Masayuki Furuya, Keisuke Uemura, Hidetoshi Hamada, Takahito Fujimori, Yu-I Hsu, Akihiro Watari, Kiyoshi Okada, Akira Myoui, Seiji Okada

**Affiliations:** 1 Present Address: Department of Orthopaedic Surgery, The University of Osaka Graduate School of Medicine, 2-2, Yamadaoka, Suita, Osaka 565-0871 Japan; 2 Department of Orthopaedic Surgery, National Hospital Organization Osaka National Hospital, 2-2, Osaka, Osaka, 540-0006 Japan; 3 Department of Applied Chemistry, The University of Osaka Graduate School of Engineering, 2-1 Yamadaoka, Suita, Osaka 565-0871 Japan; 4 Medical Center for Translational and Clinical Research, Department of Medical Innovation, The University of Osaka Hospital, 2-2, Yamadaoka, Suita, Osaka 565-0871 Japan; 5 X-innovation Committee for Health and Medical, The University of Osaka University Graduate School of Medicine, 2-2, Yamadaoka, Suita, Osaka 565- 0871 Japan

**Keywords:** Hip fracture, Osteoporosis, Hospital claim database, Second contralateral hip fractures, National database

## Abstract

**Background:**

This study aims to investigate the risk factors and onset timing of second contralateral hip fractures (SHFx) following initial femoral fractures, with a particular focus on early events occurring within one year.

**Methods:**

This retrospective study utilized a nationwide hospital claims database (Medical Data Vision, MDV) covering over 30% of all Diagnosis Procedure Combination (DPC) hospitals in Japan. MDV data combining patients who had visited a healthcare facility for osteoporosis with fractures (International Classification of Diseases, 10th Revision [ICD-10] code M80) or femoral fractures (ICD-10 code S72) were used. Patients aged 40 years and older who underwent bipolar hip arthroplasty or open reduction and internal fixation between April 2008 and September 2024 were included (*n* = 370,227). Clinical and demographic data were collected, including comorbidities and medications related to QFracture, FRAX, and fall risk.

**Results:**

The incidence of early second contralateral hip fractures (eSHFx) was 2.7% (*n* = 10,136). Multivariate logistic regression identified female, age (70–79, 80–89, ≥ 90 years), Bone Mass Index (BMI) < 18.5 kg/m2, Charlson Comorbidity Index (CCI) = 1 point, dementia, delirium, fracture history, steroid use, and sleeping pill use as significant risk factors for eSHFx. Among patients who developed SHFx, male, age ≥ 90 years, BMI < 18.5 kg/m2, CCI (1, ≥ 3 points), dementia, delirium, steroid use, and sleeping pill use were significantly associated with early rather than late onset. Fall-related factors were risk factors for eSHFx and factors that predisposed to eSHFx. Female was a risk factor for eSHFx, but the timing of SHFx was late.

**Conclusion:**

This study identified risk factors for eSHFx and factors influencing their timing. It is crucial to determine the optimal timing and method of intervention for each patient to select the most appropriate therapy.

**Supplementary Information:**

The online version contains supplementary material available at https://doi.org/10.1186/s12891-026-10160-0.

## Background

Osteoporosis is an increasingly prevalent condition in aging societies and represents a serious public health issue due to its strong association with fracture risk. A Japanese large scale cohort study involving individuals aged 40 to 74 years reported total fracture incidence rates of 7.5 per 1,000 person-years in women and 2.2 per 1,000 person-years in men [1]. Among osteoporotic fractures, hip fractures are particularly severe as they nearly always require surgical intervention and are associated with decreased mobility and increased mortality [2]. Although patients with hip fractures are commonly diagnosed with osteoporosis [3], only 10–20% of them receive appropriate treatment aimed at preventing future fractures [4, 5].

In a large prospective cohort study, second contralateral hip fractures (SHFx) were reported to occur frequently within the first year, with incidence rates ranging from 3% to 9% [6–8]. SHFx is known to further impair postoperative activities of daily living (ADL) and elevate mortality risk beyond that seen with initial fractures [7]. Therefore, early therapeutic intervention is essential for preventing SHFx. Known risk factors for SHFx include female, advanced age, weight loss, and a tendency to fall [6, 7, 9, 10]. In recent years, potent osteoporosis medications that rapidly increase bone density have become available, making it possible to tailor treatment based on SHFx risk [11]. Previous studies have shown that cases of SHFx tend to occur predominantly within the first year [7]. However, no studies have specifically investigated patient characteristics that influence the timing of SHFx occurrence. It can be inferred that patients who sustain SHFx within one year possess specific clinical characteristics.

This study aims to identify risk factors for SHFx occurring within one year and the timing of SHFx using a large-scale health insurance claims database of patients treated for femoral fractures. We hope that this study will provide new insights into the management and treatment of patients with femoral fractures, as identifying the optimal timing for more aggressive therapeutic intervention may help clarify and refine future prevention protocols.

## Methods

### Study design

This study is a retrospective study using medical insurance claims database of Medical Data Vision (MDV). The MDV database primarily includes electronic claims from DPC (Diagnosis-Related Group) hospitals. It does not include imaging data such as X-rays. DPC is a claims-based payment system managed by the Ministry of Health, Labour and Welfare, and is used in large acute care hospitals in Japan. As of December 2024, 30% of all DPC hospitals in Japan (540 facilities) were providing claim data (50 million patients) to the MDV database. MDV database includes both urban and rural large-scale hospitals, representing a broad segment of Japan’s acute care population. The MDV database includes medical data received by a patient during a specific period at a single hospital.

### Study participants

The first anonymous dataset provided by MDV included data combining patients who had visited a healthcare facility for osteoporosis with fractures (International Classification of Diseases, 10th Revision [ICD-10] code M80) or femoral fractures (ICD-10 code S72). All types of femoral fractures were included. Patients who underwent bipolar hip arthroplasty (BHA) or open reduction and internal fixation (ORIF) of hip fractures between April 2008 and September 2024 were included in this study if they were 40 years of age or older at the index date (date of the first surgery). The age threshold was set at 40 years, based on a large-scale Japanese cohort in which participants aged ≥ 40 years were followed for osteoporosis [1]. Patients who underwent two or more surgeries (BHA or ORIF) were excluded if the second surgery was performed within 14 days of the index date, as this was considered to increase the likelihood of simultaneous bilateral femoral fractures.

### Baseline demographic and clinical characteristics

For all patients, age on the index date (defined as 100 years or older in the MDV data), sex on the index date were determined. The main comorbidities and concomitant medications of interest were selected based on commonly used fracture risk assessment tools, including selected components from the QFracture [12] and FRAX tools [13], fall risk factors, and the Charlson Comorbidity Index (CCI) [14, 15], and diagnostic codes were extracted from previously published literature (Table S1). The list was verified through multiple reviews by disease area experts to ensure that the codes cover the intended conditions and medications. Factors of interest include osteoporotic fracture risk factors from QFracture and Frax tools, such as age, sex, body mass index (BMI), fracture history, steroid use, and general background factors including CCI (Acute myocardial infarction, Congestive heart failure, Peripheral vascular disease, Cerebral vascular accident, Dementia, Pulmonary disease, Connective tissue disorder, Peptic ulcer, Liver disease, Diabetes, Diabetes complications, Paraplegia, Renal disease, Cancer, Metastatic cancer, Severe liver disease, Human Immunodeficiency Virus), and fall risk factors such as dementia, delirium, and use of sleep medications. Concomitant diseases and concomitant medications were considered present if at least one instance was registered from one year prior to the index date until the discharge date of the hospitalization period including the index date. Age was categorized as < 70 years, 70–79 years, 80–89 years, and ≥ 90 years. BMI was categorized as < 18.5 kg/m^2^, 18.5–24.9 kg/m^2^, and ≥ 25.0 kg/m^2^. CCI was categorized as 0, 1, 2, and ≥ 3. Steroid use was defined as oral intake of 7.5 mg or more per day of glucocorticoid equivalents prior to surgery. All patients were eligible for analysis irrespective of postoperative osteoporosis treatment status.

### Outcome variables

Patients who have undergone two or more surgeries for BHA or ORIF of hip fractures were defined as SHFx on the contralateral side. Surgery for osteoporotic fractures of the femur is generally considered to be included in either BHA or ORIF of hip fractures. In cases of ipsilateral secondary femoral fractures, implant-related fracture codes are applied; therefore, we interpreted the definition of SHFx as not including ipsilateral secondary femoral fractures. The period up to SHFx was calculated as the number of days between the date of the second surgery and the index date. Among SHFx, those occurring within one year, which have been reported to have a high incidence rate in previous reports, were defined as early second contralateral hip fractures (eSHFx) [7], and those occurring more than one year later were defined as late second contralateral hip fractures (lSHFx). When SHFx occurs, patients are unable to walk independently, and it is expected that most patients will visit the same hospital where they underwent their initial surgery. Thus, patients not defined as SHFx were interpreted as not having SHFx regardless of the follow-up period. The flowchart is shown in Figure. First, we analyzed the background factors that cause eSHFx. Second, we analyzed the differences in background factors between eSHFx and lSHFx.

### Statistical analysis

Nine baseline factors (five osteoporosis-related fracture risk factors, one general background factor, and three fall risk factors) were used. Univariate and multivariate analyses were performed to identify predictive factors for eSHFx and risk factors for eSHFx compared to lSHFx. Multivariate analysis employed the factors of interest used in univariate analysis. The JMP statistical software version 17.0 (JMP Statistical Discovery LLC, Armonk, North Carolina, USA) was used for all statistical analyses. An overall summary, including frequencies and percentages for categorical variables, was calculated. We used the Pearson Chi-square test as appropriate to compare the measured variables between the two groups.

## Results

**Fig. 1:**
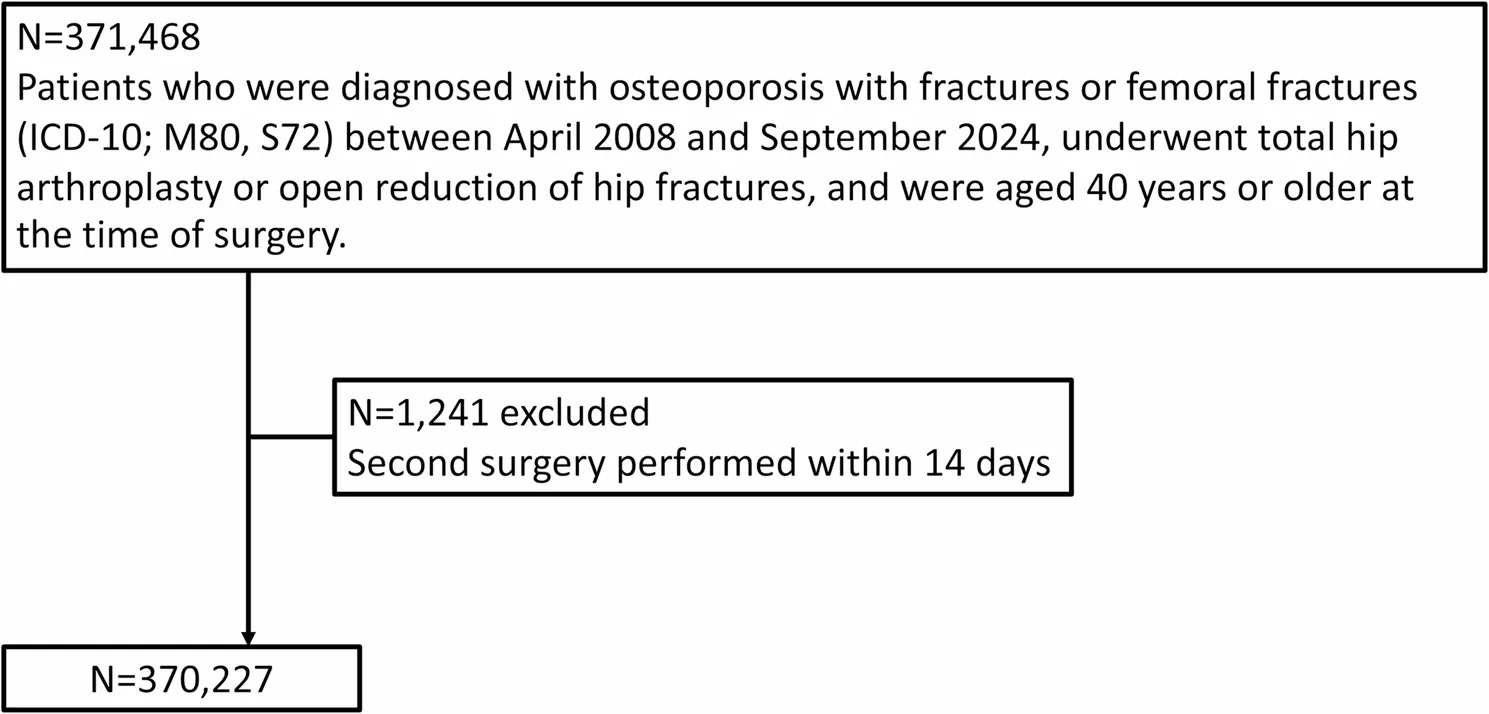
Flowchart of the study

From April 2008 to September 2024, 371,468 patients aged 40 years or older who had undergone BHA or ORIF of hip fracture and had a history of osteoporosis with fractures or femoral fractures (ICD-10; M80, S72) were identified. 1,241 cases were excluded because they had undergone a second surgery within 14 days, and 370,227 patients were finally selected as the target patients (Fig. 1). The timing of SHFx occurrence was demonstrated using a Kaplan–Meier curve, which is presented in the Supplementary Figure. Table 1 shows the baseline factors of the patients classified according to the presence or absence of early SHFx. eSHFx was significantly associated with all baseline factors. Table 2 shows the results of multivariate analysis of baseline factors in patients with and without eSHFx. In eSHFx, significant risk factors were female, age (70–79: Odds Ratio [OR] = 1.17 [1.07–1.28: 95% Confidence Interval [CI]], 80–89: OR = 1.34 [1.23–1.46: 95% CI], ≥ 90 years : OR = 1.21 [1.11–1.32: 95% CI]), BMI < 18.5 kg/m^2^ : OR = 1.16 [1.11–1.22: 95% CI], CCI = 1: OR = 1.05 [1.00–1.10: 95% CI], dementia: OR = 1.10 [1.05–1.15: 95% CI], delirium: OR = 1.15 [1.05–1.27: 95% CI], fracture history: OR = 1.19 [1.13–1.26: 95% CI], steroid use: OR = 1.30 [1.14–1.49: 95% CI], and sleeping pill use: OR = 1.11 [1.07–1.16: 95% CI]. Table 3 shows the baseline factors of patients with early or late secondary hip fracture. The rate of all baseline factors differed significantly between eSHFx and lSHFx. Table 4 shows the results of multivariate analysis of baseline factors between eSHFx and lSHFx. Age ≥ 90: OR = 1.18 [1.04–1.33: 95% CI], BMI < 18.5 kg/m^2^: OR = 1.31 [1.23–1.39: 95% CI], CCI (1: OR = 1.13 [1.05–1.20: 95% CI], ≥ 3 points: OR = 1.28 [1.02–1.62: 95% CI]), dementia: OR = 1.47 [1.37–1.57: 95% CI], delirium: OR = 1.33 [1.16–1.54: 95% CI], steroid use: OR = 1.57 [1.27–1.94: 95% CI], and sleeping pill use: OR = 1.07 [1.01–1.13: 95% CI] were significantly associated with eSHFx. Additionally, female was significantly associated with lSHFx. In the subgroup analysis of female patients, no significant differences were observed compared with the overall patient population (Table S2-S5).

**Table 1:** Characteristics of patients with or without early secondary hip fracture (eSHFx)

	Patients with eSHFx *N* = 10,136		Patients without eSHFx *N* = 360,091		*P*-value
Sex (female)	8,248	(81.4)	276,410	(76.8)	< 0.001*
Age (years)					< 0.001*
< 70	695	(6.9)	33,526	(9.3)	
70–79	1,797	(17.7)	69,600	(19.3)	
80–89	4,974	(49.1)	162,459	(45.1)	
≥ 90	2,670	(26.3)	94,506	(26.3)	
BMI (kg/m^2^)					< 0.001*
< 18.5	2,959	(31.3)	91,727	(27.6)	
18.5–24.9	5,439	(57.6)	199,853	(60.2)	
≥ 25.0	1,051	(11.1)	40,510	(12.2)	
CCI					0.005*
0	6,534	(64.6)	236,248	(66.1)	
1	2,748	(27.2)	91,579	(25.6)	
2	666	(6.5)	23,503	(6.6)	
≥ 3	172	(1.7)	5,872	(1.6)	
Dementia	3,011	(29.8)	94,432	(26.4)	< 0.001*
Delirium	505	(5.0)	14,867	(4.2)	< 0.001*
Fracture history	1,617	(16.0)	47,656	(13.3)	< 0.001*
Steroid use	231	(2.3)	6,263	(1.8)	< 0.001*
Sleeping pill use	3,633	(35.9)	117,216	(32.9)	< 0.001*

Data are presented as n (%). Study groups were compared by *Pearson Chi-square test

**Table 2:** Results of the multivariate logistic regression analysis for early secondary hip femoral (eSHFx)

Independent Variable	eSHFx		
	OR	95% CI	*p*-value
Sex (female)	1.27	1.20–1.34	< 0.001
Age (years)			
< 70	Reference		
70–79	1.17	1.07–1.28	< 0.001
80–89	1.34	1.23–1.46	< 0.001
≥ 90	1.21	1.11–1.32	< 0.001
BMI (kg/m^2^)			
< 18.5	1.16	1.11–1.22	< 0.001
18.5–24.9	Reference		
≥ 25.0	0.97	0.90–1.03	0.34
CCI (points)			
0	Reference		
1	1.05	1.00–1.10	0.03
2	1.00	0.92–1.08	0.93
≥ 3	1.04	0.89–1.22	0.61
Dementia	1.10	1.05–1.15	< 0.001
Delirium	1.15	1.05–1.27	0.003
Fracture history	1.19	1.13–1.26	< 0.001
Steroid use	1.30	1.14–1.49	< 0.001
Sleeping pill use	1.11	1.07–1.16	< 0.001

**Table 3:** Characteristics of patients with early or late secondary hip fracture (eSHFx or lSHFx)

	Patients with eSHFx *N* = 10,136		Patients with lSHFx *N* = 11,220		*P*-value
Sex (female)	8,248	(81.4)	9,508	(84.7)	< 0.001*
Age (years)					< 0.001*
< 70	695	(6.9)	789	(7.0)	
70–79	1,797	(17.7)	2,182	(19.5)	
80–89	4,974	(49.1)	5,788	(51.6)	
≥ 90	2,670	(26.3)	2,461	(21.9)	
BMI (kg/m^2^)					< 0.001*
< 18.5	2,959	(31.3)	2,685	(25.7)	
18.5–24.9	5,439	(57.6)	6,501	(62.1)	
≥ 25.0	1,051	(11.1)	1,283	(12.3)	
CCI (points)					< 0.001*
0	6,534	(64.6)	7,668	(68.4)	
1	2,748	(27.2)	2,736	(24.4)	
2	666	(6.5)	668	(6.0)	
≥ 3	172	(1.7)	146	(1.3)	
Dementia	3,011	(29.8)	2,442	(21.8)	< 0.001*
Delirium	505	(5.0)	376	(3.4)	< 0.001*
Fracture history	1,617	(16.0)	1,650	(14.7)	0.010*
Steroid use	231	(2.3)	164	(1.5)	< 0.001*
Sleeping pill use	3,633	(35.9)	3,774	(33.6)	< 0.001*

Data are presented as n (%). Study groups were compared by *Pearson Chi-square test

**Table 4:** Results of the multivariate logistic regression analysis for secondary hip femoral (eSHFx/lSHFx)

Independent Variable	eSHFx		
	OR	95% CI	*p*-value
Sex (female)	0.75	0.69–0.81	< 0.001
Age (years)			
< 70	Reference		
70–79	0.93	0.82–1.06	0.276
80–89	0.94	0.84–1.05	0.289
≥ 90	1.18	1.04–1.33	0.010
BMI			
< 18.5	1.31	1.23–1.39	< 0.001
18.5–24.9	Reference		
≥ 25.0	1.03	0.94–1.13	0.529
CCI (points)			
0	Reference		–
1	1.13	1.05–1.20	< 0.001
2	1.11	0.99–1.25	0.071
≥ 3	1.28	1.02–1.62	0.035
Dementia	1.47	1.37–1.57	< 0.001
Delirium	1.33	1.16–1.54	< 0.001
Fracture history	1.07	0.99–1.16	0.090
Steroid use	1.57	1.27–1.94	< 0.001
Sleeping pill use	1.07	1.01–1.13	0.029

On the other hand, fracture history was not significantly associated with the onset of SHFx.

## Discussion

In this study, we used a large dataset to identify significant risk factors for eSHFx and factors associated with the timing of SHFx occurrence. It has been revealed that female, age (70–79, 80–89, ≥ 90 years), BMI < 18.5 kg/m^2^, CCI = 1 point, dementia, delirium, fracture history, steroid use, and sleeping pill use are significantly associated with an increased risk of eSHFx. Among patients who experienced SHFx, male, age ≥ 90 years, BMI < 18.5 kg/m^2^, CCI (1, ≥ 3 points), dementia, delirium, steroid use, and sleeping pill use were significantly associated with eSHFx. In this study, eSHFx were primarily associated with fall-related factors rather than chronic bone fragility. This suggests that eSHFx reflects a phase of acute postoperative vulnerability characterized by transient gait instability, whereas lSHFx may represent long-term skeletal deterioration. Fall-related factors may act as acute triggers due to postoperative decline in walking function. Meanwhile, osteoporosis-related factors such as female and fracture history relating to chronic skeletal deterioration suggest an association with late-stage fractures. Compared with other countries, Japanese older adults typically have lower BMI and greater longevity [16], factors that may heighten vulnerability to both falls and bone fragility. These population differences help contextualize the patterns observed in our study.

It has been previously reported that female are at higher risk of fractures [12, 13]. In this study as well, the incidence rate of eSHFx was generally higher in female. On the other hand, among SHFx, male tended to experience eSHFx more often than female. Although this is only a speculation, possible contributing factors include delayed recovery of bone strength [17] and an increased risk of decline in activities of daily living (ADL) associated with the femoral neck [18], both of which may be related to falls.

According to the results, all fall risk factors (dementia, delirium, and use of sleep medication) were associated with an increased incidence of eSHFx, and the onset of SHFx due to fall risk factors was concentrated in the early stages. On the other hand, risk factors for osteoporotic fractures were found to increase the risk of SHFx at different times depending on the factor. Fall risk factors are considered an independent factor of osteoporotic fracture risk [19] and were found to have a significant impact on eSHFx. Appropriate rehabilitation is desired for fall prevention interventions for eSHFx [20].

The use of steroids has been reported to increase the incidence of fractures at an earlier stage and at a higher rate compared to other fracture risk factors [21]. In this study, patients who used steroids were significantly more likely to develop eSHFx with a high OR, and among patients who developed SHFx, those who used steroids had a significantly higher OR for early SHFx, indicating a substantial impact. For femoral fractures in steroid-using patients, the study suggests that careful follow-up, including early osteoporosis treatment, is necessary for secondary fractures.

It has been reported that advanced age and fracture history are at a high risk of fractures [12, 13]. In this study, it was found that age (70–79, 80–89 years) and fracture history influence the general eSHFx occurrence, but are not factors determining the timing of SHFx in those who experience re-fractures. This suggests that other factors associated with re-fracture play a more significant role in determining the timing of SHFx occurrence.

In particular, for not only female but also male, those with high fall risk, and individuals with steroid use, intensive and early anti-osteoporotic treatment is strongly recommended. Agents with rapid-onset effects on bone formation and strength, such as parathyroid hormone analogs and romosozumab, should be considered as first-line options to prevent early refracture during the vulnerable postoperative period.

This study has several limitations. The data collection was retrospective, which may introduce biases such as differences in follow-up periods and mortality rates. Since SHFx generally requires hospital admission and surgery, it is considered that patients with SHFx were appropriately evaluated. The incidence of eSHFx in this study was 2.7%, which was similar to previous reports [6–8]. Furthermore, information on bone mineral density socioeconomic status, medication adherence, and activity of daily life, which are important for assessing fracture risk, is not included in the database. To store these data, osteoporosis risk factors and fall risk factors were adopted. As this analysis relied on a hospital-based administrative database, potential coding inaccuracies or misclassifications may exist. Although we restricted our definitions to major procedural codes to minimize such errors, minor discrepancies in diagnostic coding cannot be completely excluded. In addition, there may be confounding by indication, as frailer patients are more likely to be prescribed sleeping pills, which themselves increase fall and fracture risk.

## Conclusion

In this study, we identified the risk factors associated with eSHFx, as well as the factors influencing their timing. Not only female but also male, those with high fall risk, and individuals with steroid use are greatly associated with eSHFx. Currently, various treatment options are available for osteoporosis, each with distinct efficacy and side-effect profiles. Based on these findings, it is essential to determine the optimal timing and approach of intervention for each patient and to select the most appropriate treatment accordingly. Further prospective studies are needed to confirm these findings and to establish optimal strategies for prevention of SHFx.

## Supplementary Information

Supplementary Material 1.

Supplementary Material 2.

Supplementary Material 3.

## Data Availability

The datasets generated and analyzed during the current study are available from the corresponding author upon reasonable request.

## Abbreviations

ADL: Activities of daily living
BHA: Bipolar hip arthroplasty
BMI: Body mass index
CCI: Charlson Comorbidity Index
DPC: Diagnosis Procedure Combination
eSHFx: Early second contralateral hip fractures
ICD-10: International Classification of Diseases, 10th Revision
lSHFx: Late second contralateral hip fractures
MDV: Medical Data Vision
ORIF: Open reduction and internal fixation
SHFx: Second contralateral hip fractures
