# Supplementary material for: Risk factors and early onset predictors of contralateral hip fractures following initial treatment of the other hip fracture
Source: BMC Musculoskelet Disord. 2026 Jul 2;27:789. doi: 10.1186/s12891-026-10160-0 (PMC13599093; doi:10.1186/s12891-026-10160-0)
Supplement: Supplementary file 1 [file 12891_2026_10160_MOESM2_ESM.docx]

Table S2. Characteristics of female patients with or without early secondary hip fracture (eSHFx)

|  | Patients with  eSHFx N=8,248 | | Patients without  eSHFx N=276,410 | | P-value |
| --- | --- | --- | --- | --- | --- |
| Age (years) |  |  |  |  | <0.001* |
| <70 | 407 | (4.9) | 19,378 | (4.9) |  |
| 70–79 | 1,325 | (16.1) | 48,856 | (17.7) |  |
| 80–89 | 4,142 | (50.2) | 127,412 | (46.1) |  |
| ≥90 | 2,374 | (28.8) | 80,764 | (29.2) |  |
| BMI (kg/m^2^) |  |  |  |  | <0.001* |
| <18.5 | 2,473 | (32.3) | 72,253 | (28.4) |  |
| 18.5–24.9 | 4,357 | (56.8) | 150,467 | (59.2) |  |
| ≥25.0 | 837 | (10.9) | 31,633 | (12.4) |  |
| CCI |  |  |  |  | <0.001* |
| 0 | 5,417 | (65.8) | 186,702 | (68.1) |  |
| 1 | 2,217 | (26.9) | 68,313 | (24.9) |  |
| 2 | 486 | (5.9) | 15,732 | (5.7) |  |
| ≥3 | 112 | (1.4) | 3,437 | (1.3) |  |
| Dementia | 2,603 | (31.6) | 76,890 | (28.0) | <0.001* |
| Delirium | 388 | (4.7) | 10,833 | (4.0) | <0.001* |
| Fracture history | 1,356 | (16.5) | 38,244 | (14.0) | <0.001* |
| Steroid use | 175 | (2.1) | 4,321 | (1.6) | <0.001* |
| Sleeping pill use | 2,954 | (35.9) | 90,512 | (33.1) | <0.001* |

Data are presented as n (%). Study groups were compared by *Pearson Chi-square test.

Table S3. Results of the multivariate logistic regression analysis for female early secondary hip femoral (eSHFx)

|  | eSHFx | | |
| --- | --- | --- | --- |
| Independent Variable | OR | 95% CI | p-value |
| **Age (years)** |  |  |  |
| <70 | Reference | |  |
| 70–79 | 1.25 | 1.11–1.40 | <0.001 |
| 80–89 | 1.47 | 1.32–1.63 | <0.001 |
| ≥90 | 1.32 | 1.18–1.47 | <0.001 |
| **BMI (kg/m^2^)** |  |  |  |
| <18.5 | 1.17 | 1.11–1.23 | <0.001 |
| 18.5–24.9 | Reference | |  |
| ≥25.0 | 0.93 | 0.87–1.01 | 0.08 |
| **CCI (points)** |  |  |  |
| 0 | Reference | |  |
| 1 | 1.07 | 1.02–1.13 | 0.008 |
| 2 | 1.02 | 0.92–1.12 | 0.74 |
| ≥3 | 1.07 | 0.88–1.30 | 0.49 |
| **Dementia** | 1.12 | 1.06–1.18 | <0.001 |
| **Delirium** | 1.13 | 1.01–1.26 | 0.027 |
| **Fracture history** | 1.17 | 1.10–1.25 | <0.001 |
| **Steroid use** | 1.35 | 1.15–1.58 | <0.001 |
| **Sleeping pill use** | 1.10 | 1.05–1.16 | <0.001 |

Table S4 Characteristics of female patients with early or late secondary hip fracture (eSHFx or lSHFx)

|  | Patients with  eSHFx N=8,248 | | Patients with  lSHFx N=9,508 | | P-value |
| --- | --- | --- | --- | --- | --- |
| Age (years) |  |  |  |  | <0.001* |
| <70 | 407 | (4.9) | 526 | (5.5) |  |
| 70–79 | 1,325 | (16.1) | 1,725 | (18.1) |  |
| 80–89 | 4,142 | (50.2) | 4,955 | (52.5) |  |
| ≥90 | 2,374 | (28.8) | 2,262 | (23.8) |  |
| BMI (kg/m^2^) |  |  |  |  | <0.001* |
| <18.5 | 2,473 | (32.3) | 2,322 | (26.3) |  |
| 18.5–24.9 | 4,357 | (56.8) | 5,429 | (61.4) |  |
| ≥25.0 | 837 | (10.9) | 1,093 | (12.4) |  |
| CCI (points) |  |  |  |  | <0.001* |
| 0 | 5,417 | (65.8) | 6,613 | (69.6) |  |
| 1 | 2,217 | (26.9) | 2,259 | (23.8) |  |
| 2 | 486 | (5.9) | 528 | (5.6) |  |
| ≥3 | 112 | (1.4) | 106 | (1.1) |  |
| Dementia | 2,603 | (31.6) | 2,175 | (22.9) | <0.001* |
| Delirium | 388 | (4.7) | 313 | (3.3) | <0.001* |
| Fracture history | 1,356 | (16.5) | 1,436 | (15.1) | 0.013* |
| Steroid use | 175 | (2.1) | 132 | (1.4) | <0.001* |
| Sleeping pill use | 3,633 | (35.9) | 3,774 | (33.6) | <0.001* |

Data are presented as n (%). Study groups were compared by *Pearson Chi-square test.

Table S5 Results of the multivariate logistic regression analysis for　female secondary hip femoral (eSHFx/lSHFx)

|  | eSHFx | | |
| --- | --- | --- | --- |
| Independent Variable | OR | 95% CI | p-value |
| **Age (years)** |  |  |  |
| <70 | Reference | |  |
| 70–79 | 0.95 | 0.82–1.11 | 0.530 |
| 80–89 | 0.97 | 0.84–1.12 | 0.644 |
| ≥90 | 1.20 | 1.03–1.39 | 0.019 |
| **BMI** |  |  |  |
| <18.5 | 1.30 | 1.21–1.40 | <0.001 |
| 18.5–24.9 | Reference | |  |
| ≥25.0 | 1.00 | 0.91–1.11 | 0.952 |
| **CCI (points)** |  |  |  |
| 0 | Reference | | – |
| 1 | 1.15 | 1.07–1.24 | <0.001 |
| 2 | 1.09 | 0.95–1.24 | 0.217 |
| ≥3 | 1.27 | 0.96–1.68 | 0.091 |
| **Dementia** | 1.48 | 1.38–1.59 | <0.001 |
| **Delirium** | 1.28 | 1.09–1.51 | 0.002 |
| **Fracture history** | 1.07 | 0.98–1.16 | 0.137 |
| **Steroid use** | 1.60 | 1.26–2.02 | <0.001 |
| **Sleeping pill use** | 1.07 | 1.01–1.13 | 0.029 |
