# Supplementary material for: Risk factors and early onset predictors of contralateral hip fractures following initial treatment of the other hip fracture
Source: BMC Musculoskelet Disord. 2026 Jul 2;27:789. doi: 10.1186/s12891-026-10160-0 (PMC13599093; doi:10.1186/s12891-026-10160-0)
Supplement: Supplementary file 2 [file 12891_2026_10160_MOESM3_ESM.docx]

Table. S1

ICD-10 code of variable

| Variable | Code |
| --- | --- |
| Charlson comorbidity index | I21, I22, I252, I50, I71, I790, I739, R02, Z958, Z959, G450, G451, G452, G458, G459, G46, I64, G454, I670, I671, I672, I674, I675, I676, I677, I678, I679, I681, I682, I688, I690, I691, I693, I694, F00, F01, F02, F051, J40, J41, J42, J44, J43, J45, J46, J47, J67, J60, J61, J62, J63, J66, J64, J65, M32, M34, M332, M053, M058, M059, M060, M063, M069, M050, M052, M051, M353, K25, K26, K27, K28, K702, K703, K73, K717, K740, K742, K746, K743, K744, K745, E109, E119, E139, E149, E101, E111, E131, E141, E105, E115, E135, E145, E102, E112, E132, E142, E103, E113, E133, E143, E104, E114, E134, E144, G810, G811, G819, G041, G820, G821, G822, N03, N052, N053, N054, N055, N056, N072, N073, N074, N01, N18, N19, N25, C0, C1, C2, C3, C40, C41, C43, C45, C46, C47, C48, C49, C5, C6, C70, C71, C72, C73, C74, C75, C76, C80, C81, C82, C83, C84, C85, C883, C887, C889, C900, C901, C91, C92, C93, C940, C941, C942, C943, C9451, C947, C95, C96, C77, C78, C79, C80, K729, K766, K767, K721, B20, B21, B22, B23, B24 |
| Dementia | F010, F011, F012, F019, F03, G300, G301, G308, G309, G310, G318 |
| Delirium | F059, F051, F050 |
| Fracture history | S2230, S2231, S2240, S2241, S3210, S3211, S3250, S3251, S4220, S4221, S5250, S5251, S5260, S5261, S5290, S5291, S8210, S8211, S8230, S8231, S8240, S8241, T08, S2200, S2201, S2210, S2211, S320, S3200, S3201 |
